# Supplementary material for: Social Participation and Functional Decline: A Comparative Study of Rural and Urban Older People, Using Japan Gerontological Evaluation Study Longitudinal Data
Source: Int J Environ Res Public Health. 2020 Jan 18;17(2):617. doi: 10.3390/ijerph17020617 (PMC7013913; doi:10.3390/ijerph17020617)
Supplement: Supplementary file 1 [file ijerph-17-00617-s001.pdf]

International Journal of
Environmental Research and Public Health

MDPI

Table S1. Complete case analysis: HRs for participation in one, two, and three or more different types of organizations.

**Rural**	**Crude model**	**Model 1**	**Model 2**
n=5,673	HR (95% CI)	HR (95% CI)	HR (95% CI)
0	1.00 Ref	1.00 Ref	1.00 Ref
1	0.58* (0.47–0.71)	0.81 (0.66–1.001)	0.88 (0.72–1.09)
2	0.46* (0.37–0.58)	0.69* (0.55–0.87)	0.77* (0.61–0.97)
≥3	0.36* (0.30–0.43)	0.59* (0.48–0.72)	0.72* (0.58–0.90)
Trend P	$p < 0.05$	$p < 0.05$	$p < 0.05$
Urban	Crude model	Model 1	Model 2
n=13,357	HR (95% CI)	HR (95% CI)	HR (95% CI)
0	1.00 Ref	1.00 Ref	1.00 Ref
1	0.57* (0.50–0.66)	0.75* (0.65–0.86)	0.81* (0.85–0.94)
2	0.53* (0.46–0.62)	0.75* (0.65–0.87)	0.86* (0.74–0.99)
≥3	0.41* (0.36–0.47)	0.61* (0.53–0.70)	0.77* (0.67–0.89)
Trend P	$p < 0.05$	$p < 0.05$	$p < 0.05$

HR: Hazard ratio; CI: confidence interval; Ref: reference. *$p < 0.05$.

Model 1: Crude model + sex, age, equivalent income, educational attainment, marital status, and self-reported medical conditions. Model 2: Model 1 + smoking, alcohol consumption, walking time (per. day), frequency of going outdoors, depression, emotional support, instrumental support, frequency of meeting friends, and IADL.

Table S2. The full modeling results for participation in one, two, and three or more different types of organizations in Model 2.

	Rural	Urban
	HR (95% CI)	HR (95% CI)
Number of types of organizations (ref. 0)		
1	0.94 (0.84–1.05)	0.92* (0.85–0.99)
2	0.85* (0.75–0.97)	0.87* (0.80–0.96)
≥3	0.76* (0.67–0.86)	0.82* (0.75–0.89)
Missing	0.99 (0.82–1.20)	0.95 (0.82–1.10)
Sex (ref. male)		
Female	1.14* (1.01–1.29)	1.02 (0.94–1.11)
Age (ref. 65-69)		
70–74	1.85* (1.57–2.19)	2.09* (1.87–2.34)
75–79	3.59* (3.07–4.21)	4.37* (3.92–4.87)
80–84	7.24* (6.16–8.50)	8.26* (7.38–9.24)
85+	11.73* (9.83–14.00)	12.70* (11.19–14.42)
Equivalent income (ref. low)		
Middle	0.87* (0.78–0.97)	1.02 (0.95–1.09)
High	1.04 (0.87–1.25)	0.89* (0.79–0.99)
Missing	0.89* (0.80–0.98)	1.02 (0.94–1.10)
Educational attainment (ref. < 10 years)		
10–12	0.89* (0.80–0.98)	1.06 (0.95–1.09)
≥ 13	1.02 (0.88–1.17)	1.02 (0.79–0.99)
Missing	1.16* (0.95–1.41)	1.12 (0.94–1.10)
Marital status (ref. married)		
Single	1.08 (0.98–1.19)	1.08* (1.01–1.16)
Missing	1.11 (0.88–1.39)	1.12 (0.96–1.32)
Self-reported medical conditions (ref. illness)		
No illness	0.76* (0.67–0.85)	0.75* (0.69–0.82)
Missing	0.95 (0.83–1.09)	0.98 (0.89–1.07)
Smoking (ref. never smoked)		
Past smoker	1.20* (1.06–1.36)	1.03 (0.94–1.12)
Current smoker	1.47* (1.25–1.73)	1.28* (1.15–1.43)
Missing	1.01 (0.88–1.17)	1.00 (0.89–1.11)
Alcohol consumption (ref. current drinker)		
Past drinker	1.28* (1.03–1.59)	1.25* (1.07–1.45)
Never drank	0.98 (0.87–1.09)	1.11* (1.03–1.19)
Missing	1.09 (0.87–1.36)	1.13* (0.95–1.35)
Walking time (ref. <30 min/day)		
30–60	0.85* (0.77–0.94)	0.87* (0.81–0.93)
60–90	0.80* (0.70–0.92)	0.80* (0.73–0.88)
>90	0.73* (0.63–0.83)	0.73* (0.66–0.81)
Missing	0.97 (0.82–1.14)	0.84* (0.74–0.96)
Frequency of going outdoors (ref. almost every day)		
2–3 times/week	1.16* (1.05–1.29)	1.11* (1.03–1.18)
About once/week	1.10 (0.96–1.26)	1.09 (0.99–1.22)
Rarely	1.14* (0.99–1.31)	1.34* (1.19–1.49)
Missing	1.11 (0.91–1.35)	1.18 (0.99–1.40)
Depression (ref. no depression)		
Depressive tendency	1.19* (1.07–1.33)	1.12* (1.16–1.34)
Depression	1.41* (1.20–1.64)	1.35* (1.20–1.51)

Missing	1.06* (1.04–1.30)	1.15* (1.07–1.25)
Emotional support (ref. available)		
Not available	1.15* (1.02–1.30)	1.12* (1.03–1.23)
Missing	0.95 (0.75–1.21)	0.93 (0.76–1.16)
Instrumental support (ref. available)		
Not available	1.10 (0.99–1.23)	1.19* (1.10–1.28)
Missing	1.08 (0.84–1.37)	1.15 (0.93–1.42)
Frequency of meeting friends (ref. almost every day)		
2–3 times/week	0.97 (0.84–1.11)	1.01 (0.91–1.12)
About once/week	1.00 (0.85–1.16)	1.00 (0.89–1.11)
1–2 times/month	1.01 (0.87–1.18)	1.01 (0.90–1.12)
A few times a year or less	0.96 (0.83–1.12)	1.01 (0.91–1.13)
Missing	1.04 (0.87–1.26)	1.05 (0.91–1.22)
IADL (ref. not decline)		
Decline	1.27* (1.44–1.40)	1.21* (1.13–1.30)
Missing	1.41* (1.23–1.62)	1.19* (1.07–1.32)

HR: Hazard ratio; CI: confidence interval; Ref: reference. *p < 0.05.

Model 1: Crude model + sex, age, equivalent income, educational attainment, marital status, and self-reported medical conditions. Model 2: Model 1 + smoking, alcohol consumption, walking time (per. day), frequency of going outdoors, depression, emotional support, instrumental support, frequency of meeting friends, and IADL.

Table S3. Complete case analysis: HRs for type of social participation (reference: nonparticipation in each organization).

**Rural**	**Crude model**	**Model 1**	**Model 2**
n=5,673	HR (95% CI)	HR (95% CI)	HR (95% CI)
Local community	0.58* (0.50–0.68)	0.82* (0.70–0.96)	0.89 (0.76–1.04)
Hobby	0.51* (0.43–0.60)	0.65* (0.55–0.77)	0.71* (0.60–0.85)
Sports	0.52* (0.42–0.63)	0.63* (0.51–0.78)	0.70* (0.56–0.86)
Industry	0.59* (0.47–0.73)	0.81 (0.64–1.02)	0.92 (0.73–1.16)
Volunteer	0.63* (0.51–0.78)	0.80* (0.65–0.99)	0.93 (0.75–1.16)
Citizen	1.26* (1.08–1.48)	0.90 (0.77–1.06)	0.99 (0.84–1.17)
Work	0.47* (0.38–0.57)	0.75* (0.61–0.92)	0.85 (0.68–1.05)
Urban	Crude model	Model 1	Model 2
n=13,357	HR (95% CI)	HR (95% CI)	HR (95% CI)
Local community	0.68* (0.61–0.75)	0.82* (0.74–0.91)	0.91 (0.82–1.02)
Hobby	0.62* (0.56–0.69)	0.75* (0.68–0.84)	0.88* (0.79–0.99)
Sports	0.57* (0.50–0.64)	0.74* (0.65–0.84)	0.87*(0.75–0.99)
Industry	0.74* (0.64–0.85)	0.89 (0.78–1.03)	1.03 (0.89–1.19)
Volunteer	0.60* (0.52–0.70)	0.76* (0.65–0.89)	0.91 (0.78–1.07)
Citizen	1.28* (1.14–1.44)	0.86* (0.77–0.97)	0.96 (0.85–1.09)
Work	0.48* (0.42–0.55)	0.78*(0.68–0.90)	0.82* (0.71–0.95)

HR: Hazard ratio; CI: confidence interval; Ref: reference. *p < 0.05.

Model 1: Crude model + sex, age, equivalent income, educational attainment, marital status, and self-reported medical conditions. Model 2: Model 1 + smoking, alcohol consumption, walking time (per. day), frequency of going outdoors, depression, emotional support, instrumental support, frequency of meeting friends, and IADL.

International Journal of
Environmental Research and Public Health

MDPI

Table S4. The full modeling results for HRs for type of social participation (reference: nonparticipation in each organization).

**Rural**	**Local community**	**Hobby**	**Sports**	**Industry**	**Volunteer**	**Citizen**	**Work**
	HR (95% CI)	HR (95% CI)	HR (95% CI)	HR (95% CI)	HR (95% CI)	HR (95% CI)	HR (95% CI)
Types of social participation (ref. nonparticipation)							
Participation	0.86* (0.77–0.95)	0.76* (0.68–0.85)	0.79* (0.69–0.89)	1.01* (0.87–1.18)	0.89 (0.77–1.03)	1.02 (0.93–1.13)	0.80* (0.70–0.91)
Missing	1.00 (0.89–1.11)	0.96 (0.86–1.07)	0.98 (0.88–1.09)	1.04 (0.94–1.14)	1.07 (0.98–1.18)	1.08 (0.97–1.21)	0.97 (0.88–1.08)
Sex (ref. male)							
Female	1.15* (1.02–1.30)	1.17* (1.04–1.32)	1.16* (1.03–1.31)	1.17* (1.03–1.32)	1.16* (1.03–1.31)	1.17* (1.03–1.32)	1.15* (1.01–1.30)
Age (ref. 65-69)							
70–74	1.85* (1.57–2.18)	1.87* (1.58–2.20)	1.87* (1.58–2.20)	1.85* (1.57–2.18)	1.84* (1.56–2.18)	1.84* (1.56–2.18)	1.81* (1.53–2.14)
75–79	3.57* (3.05–4.19)	3.62* (3.09–4.24)	3.63* (3.10–4.26)	3.59* (3.06–4.21)	3.56* (3.04–4.18)	3.57* (3.04–4.20)	3.47* (2.95–4.07)
80–84	7.20* (6.13–8.46)	7.27* (6.19–8.54)	7.33* (6.24–8.60)	7.26* (6.18–8.54)	7.19* (6.12–8.45)	7.24* (6.15–8.52)	6.99* (5.94–8.22)
85+	11.65* (9.76–13.91)	11.86* (9.93–14.16)	11.94* (10.00–14.25)	11.89* (9.96–14.20)	11.75* (9.84–14.03)	11.83* (9.90–14.15)	11.40* (9.53–13.63)
Equivalent income (ref. low)							
Middle	0.87* (0.78–0.97)	0.87* (0.78–0.98)	0.87* (0.78–0.97)	0.87* (0.78–0.97)	0.87* (0.78–0.98)	0.87* (0.78–0.97)	0.87* (0.78–0.97)
High	1.02 (0.85–1.22)	1.04 (0.86–1.24)	1.03 (0.85–1.23)	1.03 (0.86–1.24)	1.03 (0.86–1.24)	1.03 (0.86–1.24)	1.05 (0.88–1.27)
Missing	0.89* (0.80–0.99)	0.89* (0.81–0.99)	0.90* (0.81–0.967)	0.90* (0.81–0.99)	0.89* (0.81–0.99)	0.90 (0.81–0.99)	0.90 (0.81–1.00)
Educational attainment (ref. < 10 years)							
10–12	0.88* (0.79–0.97)	0.89* (0.81–0.99)	0.88* (0.79–0.97)	0.87* (0.79–0.97)	0.88* (0.79–0.97)	0.87* (0.79–0.97)	0.87* (0.79–0.96)
≥ 13	1.01 (0.87–1.16)	1.03 (0.89–1.19)	1.01 (0.88–1.17)	1.00 (0.87–1.16)	1.01 (0.88–1.17)	1.00 (0.87–1.16)	0.99 (0.86–1.15)
Missing	1.15* (0.95–1.41)	1.16 (0.95–1.41)	1.15* (0.94–1.41)	1.15 (0.95–1.41)	1.16 (0.95–1.41)	1.16 (0.95–1.41)	1.16 (0.95–1.42)
Marital status (ref. married)							
Single	1.08 (0.98–1.20)	1.08 (0.98–1.19)	1.08 (0.98–1.19)	1.08 (0.98–1.19)	1.08 (0.98–1.20)	1.08 (0.98–1.20)	1.07 (0.97–1.18)
Missing	1.12 (0.89–1.40)	1.12 (0.89–1.40)	1.12 (0.89–1.40)	1.12 (0.89–1.40)	1.12 (0.89–1.41)	1.12 (0.89–1.40)	1.11 (0.88–1.39)
Self-reported medical conditions (ref. illness)							
No illness	0.76* (0.67–0.85)	0.75* (0.67–0.85)	0.75* (0.67–0.85)	0.75* (0.67–0.85)	0.76* (0.67–0.85)	0.76* (0.67–0.85)	0.76* (0.68–0.85)
Missing	0.95 (0.83–1.08)	0.94 (0.83–1.08)	0.95 (0.83–1.09)	0.94 (0.83–1.08)	0.95 (0.83–1.08)	0.95 (0.83–1.08)	0.95 (0.83–1.09)
Smoking (ref. never smoked)							
Past smoker	1.22* (1.07–1.38)	1.21* (1.07–1.37)	1.22* (1.07–1.38)	1.22* (1.07–1.38)	1.22* (1.07–1.38)	1.21* (1.07–1.38)	1.21* (1.06–1.37)
Current smoker	1.49* (1.27–1.75)	1.48* (1.26–1.74)	1.48* (1.26–1.74)	1.49* (1.27–1.75)	1.49* (1.26–1.74)	1.49* (1.27–1.75)	1.49* (1.26–1.75)
Missing	1.01 (0.88–1.17)	1.02 (0.88–1.17)	1.02 (0.88–1.18)	1.02 (0.88–1.17)	1.01 (0.88–1.17)	1.02 (0.88–1.17)	1.02 (0.88–1.18)
Alcohol consumption (ref. current drinker)							
Past drinker	1.29* (1.04–1.61)	1.29* (1.04–1.61)	1.29* (1.03–1.60)	1.30* (1.04–1.62)	1.30* (1.04–1.62)	1.30* (1.04–1.62)	1.29* (1.04–1.61)
Never drank	0.99 (0.88–1.10)	0.98 (0.88–1.09)	0.98 (0.87–1.09)	0.99 (0.89–1.11)	0.99 (0.89–1.11)	0.99 (0.89–1.11)	0.99 (0.89–1.10)

Missing	1.09 (0.87–1.35)	1.08 (0.88–1.35)	1.07 (0.86–1.34)	1.09 (0.87–1.36)	1.09 (0.88–1.36)	1.09 (0.88–1.36)	1.08 (0.87–1.35)
Walking time (ref. <30 min/day)							
30–60	0.85* (0.77–0.94)	0.85* (0.77–0.94)	0.85* (0.77–0.94)	0.85* (0.77–0.94)	0.85* (0.77–0.94)	0.85* (0.77–0.94)	0.84* (0.76–0.93)
60–90	0.80* (0.70–0.91)	0.80* (0.70–0.92)	0.80* (0.70–0.92)	0.79* (0.69–0.91)	0.79* (0.69–0.91)	0.79* (0.69–0.91)	0.79* (0.69–0.91)
>90	0.72* (0.62–0.82)	0.72* (0.62–0.82)	0.72* (0.62–0.83)	0.71* (0.62–0.82)	0.71* (0.62–0.82)	0.71* (0.62–0.82)	0.73* (0.64–0.84)
Missing	0.98 (0.83–1.16)	0.98 (0.83–1.16)	0.99 (0.83–1.17)	0.98 (0.83–1.16)	0.98 (0.83–1.15)	0.98 (0.83–1.16)	1.00 (0.84–1.18)
Frequency of going outdoors (ref. almost every day)							
2–3 times/week	1.17* (1.06–1.30)	1.17* (1.05–1.30)	1.17* (1.05–1.29)	1.17* (1.06–1.30)	1.17* (1.05–1.30)	1.17* (1.06–1.30)	1.16* (1.05–1.29)
About once/week	1.12 (0.98–1.28)	1.11 (0.97–1.27)	1.11 (0.97–1.27)	1.13 (0.99–1.29)	1.12 (0.98–1.28)	1.13 (0.98–1.29)	1.13 (0.99–1.29)
Rarely	1.16* (1.01–1.33)	1.14* (1.00–1.31)	1.15* (1.01–1.33)	1.17* (1.02–1.35)	1.17* (1.02–1.34)	1.18* (1.02–1.35)	1.18* (1.02–1.35)
Missing	1.12 (0.92–1.37)	1.12 (0.92–1.37)	1.12 (0.92–1.37)	1.13 (0.93–1.38)	1.13 (0.93–1.37)	1.13 (0.93–1.38)	1.13 (0.93–1.38)
Depression (ref. no depression)							
Depressive tendency	1.20* (1.08–1.34)	1.29* (1.07–1.32)	1.20* (1.08–1.33)	1.21* (1.08–1.35)	1.20* (1.08–1.34)	1.21* (1.08–1.35)	1.20* (1.08–1.34)
Depression	1.44* (1.23–1.68)	1.42* (1.21–1.66)	1.43* (1.22–1.67)	1.45* (1.24–1.70)	1.44* (1.23–1.69)	1.45* (1.24–1.69)	1.43* (1.23–1.68)
Missing	1.17* (1.04–1.31)	1.17* (1.05–1.31)	1.17* (1.05–1.31)	1.18* (1.05–1.32)	1.17* (1.05–1.31)	1.17* (1.05–1.31)	1.18* (1.05–1.32)
Emotional support (ref. available)							
Not available	1.15* (1.02–1.31)	1.15* (1.02–1.31)	1.15* (1.02–1.31)	1.16* (1.02–1.31)	1.15* (1.02–1.30)	1.16* (1.02–1.31)	1.16* (1.02–1.31)
Missing	0.96 (0.75–1.22)	0.95 (0.75–1.21)	0.96 (0.76–1.22)	0.96 (0.75–1.21)	0.95 (0.75–1.21)	0.96 (0.75–1.21)	0.96 (0.76–1.23)
Instrumental support (ref. available)							
Not available	1.11* (1.02–1.31)	1.11 (1.00–1.24)	1.11 (1.00–1.24)	1.12* (1.002–1.25)	1.12 (1.00–1.25)	1.12* (1.001–1.25)	1.12* (1.005-1.25)
Missing	1.09 (0.75–1.22)	1.09 (0.85–1.39)	1.09 (0.86–1.39)	1.09 (0.86–1.39)	1.08 (0.85–1.38)	1.09 (0.85–1.38)	1.09 (0.86–1.39)
Frequency of meeting friends (ref. almost every day)							
2–3 times/week	0.95 (0.82–1.09)	0.97 (0.84–1.12)	0.96 (0.83–1.11)	0.95 (0.82–1.09)	0.95 (0.82–1.10)	0.95 (0.82–1.09)	0.94 (0.81–1.08)
About once/week	0.98 (0.84–1.15)	1.01 (0.86–1.18)	0.99 (0.85–1.15)	0.98 (0.84–1.15)	0.99 (0.85–1.16)	0.99 (0.84–1.15)	0.97 (0.83–1.14)
1–2 times/month	1.01 (0.87–1.17)	1.01 (0.87–1.18)	1.00 (0.86–1.16)	1.00 (0.87–1.17)	1.01 (0.87–1.17)	1.01 (0.87–1.17)	1.00 (0.86–1.15)
A few times a year or less	0.99 (0.85–1.15)	0.97 (0.83–1.12)	0.97 (0.84–1.13)	1.00 (0.86–1.16)	1.00 (0.86–1.16)	1.00 (0.86–1.17)	0.98 (0.85–1.14)
Missing	1.07 (0.88–1.29)	1.08 (0.89–1.30)	1.08 (0.89–1.30)	1.07 (0.89–1.30)	1.06 (0.88–1.28)	1.06 (0.88–1.28)	1.08 (0.90–1.30)
IADL (ref. not decline)							
Decline	1.29* (1.17–1.43)	1.27* (1.14–1.40)	1.29* (1.17–1.43)	1.31* (1.18–1.45)	1.30* (1.18–1.44)	1.31* (1.18–1.45)	1.30* (1.18–1.44)
Missing	1.44* (1.25–1.66)	1.41* (1.23–1.63)	1.44* (1.25–1.66)	1.46* (1.27–1.68)	1.45* (1.26–1.66)	1.46* (1.27–1.68)	1.45* (1.26–1.67)

**Urban**	**Local community**	**Hobby**	**Sports**	**Industry**	**Volunteer**	**Citizen**	**Work**
	HR (95% CI)	HR (95% CI)	HR (95% CI)	HR (95% CI)	HR (95% CI)	HR (95% CI)	HR (95% CI)
Types of social participation (ref. nonparticipation)							

Participation	0.95 (0.88–1.01)	0.90* (0.84–0.97)	0.83* (0.77–0.91)	1.04 (0.95–1.15)	0.94 (0.85–1.04)	0.99 (0.92–1.07)	0.83* (0.76–0.91)
Missing	1.00 (0.93–1.07)	0.99 (0.92–1.08)	0.95 (0.88–1.03)	1.04 (0.97–1.12)	1.03 (0.97–1.11)	1.05 (0.97–1.13)	0.98 (0.90–1.06)
Sex (ref. male)							
Female	1.03 (0.94–1.12)	1.03 (0.95–1.13)	1.04 (0.95–1.13)	1.04 (0.95–1.13)	1.03 (0.95–1.12)	1.03 (0.95–1.13)	1.02 (0.94–1.11)
Age (ref. 65-69)							
70–74	2.10* (1.87–2.35)	2.10* (1.88–2.35)	2.10* (1.87–2.35)	2.09* (1.87–2.34)	2.09* (1.87–2.34)	2.10* (1.87–2.35)	2.06* (1.84–2.31)
75–79	4.38* (3.93–4.88)	4.40* (3.95–4.90)	4.40* (3.95–4.90)	4.38* (3.92–4.87)	4.37* (3.92–4.87)	4.38* (3.93–4.89)	4.26* (3.82–4.75)
80–84	8.33* (7.45–9.32)	8.35* (7.47–9.34)	8.32* (7.44–9.31)	8.33* (7.44–9.32)	8.31* (7.42–9.29)	8.35* (7.46–9.35)	8.07* (7.20–9.04)
85+	12.82* (11.30–14.56)	12.84* (11.32–14.58)	12.81* (11.39–14.54)	12.85* (11.32–14.59)	12.81* (11.28–14.54)	12.91* (11.37–14.67)	12.45* (10.96–14.15)
Equivalent income (ref. low)							
Middle	1.01 (0.94–1.08)	1.01 (0.94–1.09)	1.01 (0.94–1.09)	1.01 (0.94–1.08)	1.01 (0.94–1.08)	1.01 (0.94–1.08)	1.01 (0.94–1.08)
High	0.87* (0.78–0.97)	0.88* (0.79–0.98)	0.88* (0.79–0.98)	0.87* (0.78–0.97)	0.88* (0.79–0.98)	0.88* (0.79–0.97)	0.90* (0.80–0.998)
Missing	1.02 (0.94–1.10)	1.02 (0.94–1.10)	1.02 (0.94–1.10)	1.02 (0.94–1.10)	1.02 (0.94–1.10)	1.02 (0.94–1.10)	1.02 (0.94–1.10)
Educational attainment (ref. < 10 years)							
10–12	1.05 (0.99–1.13)	1.06 (1.00–1.14)	1.06 (0.99–1.13)	1.05 (0.99–1.12)	1.06 (0.99–1.13)	1.05 (0.99–1.12)	1.05 (0.98–1.12)
≥ 13	1.01 (0.93–1.10)	1.03 (0.94–1.12)	1.02 (0.94–1.12)	1.01 (0.93–1.10)	1.02 (0.93–1.11)	1.01 (0.93–1.10)	1.01 (0.93–1.10)
Missing	1.13 (0.96–1.32)	1.13 (0.96–1.33)	1.13 (0.96–1.33)	1.13 (0.96–1.32)	1.13 (0.96–1.33)	1.13 (0.96–1.32)	1.13 (0.96–1.33)
Marital status (ref. married)							
Single	1.08* (1.01–1.16)	1.08* (1.01–1.16)	1.08* (1.01–1.15)	1.08* (1.01–1.16)	1.08* (1.01–1.16)	1.08* (1.01–1.16)	1.08* (1.01–1.16)
Missing	1.01 (0.82–1.25)	1.01 (0.82–1.25)	1.01 (0.82–1.24)	1.01 (0.82–1.25)	1.01 (0.82–1.25)	1.01 (0.82–1.25)	1.02 (0.96–1.26)
Self-reported medical conditions (ref. illness)							
No illness	0.75* (0.69–0.81)	0.75* (0.69–0.81)	0.75* (0.69–0.81)	0.75* (0.69–0.81)	0.75* (0.69–0.81)	0.75* (0.69–0.81)	0.75* (0.70–0.82)
Missing	0.98 (0.89–1.08)	0.98 (0.89–1.08)	0.98 (0.89–1.08)	0.98 (0.89–1.08)	0.98 (0.89–1.08)	0.98 (0.89–1.08)	0.98 (0.89–1.08)
Smoking (ref. never smoked)							
Past smoker	1.03 (0.95–1.13)	1.03 (0.95–1.13)	1.04 (0.95–1.13)	1.03 (0.95–1.13)	1.03 (0.94–1.13)	1.03 (0.94–1.13)	1.03 (0.94–1.12)
Current smoker	1.29* (1.16–1.44)	1.28* (1.15–1.44)	1.29* (1.15–1.44)	1.29* (1.16–1.44)	1.29* (1.15–1.44)	1.29* (1.16–1.44)	1.29* (1.16–1.45)
Missing	1.00 (0.90–1.12)	1.00 (0.89–1.12)	1.00 (0.90–1.12)	1.00 (0.89–1.12)	1.00 (0.89–1.12)	1.00 (0.89–1.12)	1.00 (0.90–1.12)
Alcohol consumption (ref. current drinker)							
Past drinker	1.26* (1.08–1.47)	1.25* (1.07–1.46)	1.25* (1.07–1.45)	1.26* (1.08–1.47)	1.26* (1.08–1.47)	1.26* (1.08–1.47)	1.25* (1.07–1.45)
Never drank	1.11* (1.03–1.20)	1.11* (1.03–1.20)	1.11* (1.03–1.19)	1.12* (1.04–1.21)	1.12* (1.04–1.20)	1.12* (1.04–1.20)	1.11* (1.03–1.20)
Missing	1.13 (0.94–1.34)	1.13 (0.95–1.35)	1.12 (0.94–1.34)	1.13 (0.94–1.34)	1.13 (0.94–1.34)	1.13* (0.94–1.34)	1.12* (0.94–1.34)
Walking time (ref. <30 min/day)							
30–60	0.86* (0.81–0.92)	0.86* (0.81–0.92)	0.87* (0.81–0.93)	0.86* (0.80–0.92)	0.86* (0.81–0.92)	0.86* (0.81–0.92)	0.86* (0.80–0.92)
60–90	0.80* (0.73–0.87)	0.80* (0.73–0.87)	0.80* (0.73–0.88)	0.79 (0.72–0.87)	0.80* (0.72–0.87)	0.79* (0.72–0.87)	0.80* (0.73–0.87)
>90	0.72* (0.65–0.80)	0.72* (0.65–0.80)	0.73* (0.66–0.81)	0.72* (0.65–0.80)	0.72* (0.65–0.80)	0.72* (0.65–0.80)	0.74* (0.67–0.82)
Missing	0.84* (0.74–0.96)	0.84* (0.74–0.96)	0.85* (0.74–0.97)	0.84* (0.73–0.96)	0.84* (0.73–0.96)	0.84* (0.73–0.96)	0.85* (0.74–0.97)

Frequency of going outdoors (ref. almost every day)							
2–3 times/week	1.11* (1.04–1.19)	1.11* (1.04–1.19)	1.10* (1.03–1.18)	1.11* (1.04–1.19)	1.11* (1.03–1.19)	1.11* (1.04–1.19)	1.10* (1.03–1.18)
About once/week	1.11 (1.00–1.23)	1.10 (0.99–1.22)	1.10 (0.99–1.22)	1.11 (1.00–1.23)	1.11 (1.00–1.22)	1.11 (1.00–1.23)	1.11 (1.00–1.23)
Rarely	1.35* (1.21–1.51)	1.34* (1.20–1.50)	1.34* (1.20–1.50)	1.35* (1.21–1.51)	1.35* (1.21–1.51)	1.35* (1.21–1.51)	1.35* (1.21–1.51)
Missing	1.18 (1.00–1.41)	1.18 (1.00–1.41)	1.18 (0.99–1.40)	1.19 (1.00–1.41)	1.19 (1.00–1.41)	1.19 (1.00–1.41)	1.18 (1.00–1.41)
Depression (ref. no depression)							
Depressive tendency	1.26* (1.17–1.36)	1.26* (1.16–1.35)	1.26* (1.17–1.36)	1.26* (1.17–1.36)	1.26* (1.17–1.36)	1.26* (1.17–1.36)	1.26* (1.17–1.36)
Depression	1.37* (1.22–1.54)	1.36* (1.21–1.53)	1.37* (1.22–1.54)	1.38* (1.23–1.55)	1.38* (1.23–1.55)	1.38* (1.23–1.55)	1.37* (1.22–1.54)
Missing	1.16* (1.07–1.25)	1.16* (1.07–1.25)	1.16* (1.08–1.26)	1.16* (1.07–1.25)	1.16* (1.07–1.25)	1.16* (1.07–1.25)	1.16* (1.07–1.25)
Emotional support (ref. available)							
Not available	1.13* (1.10–1.29)	1.13* (1.03–1.24)	1.13* (1.03–1.25)	1.13* (1.03–1.24)	1.13* (1.03–1.24)	1.13* (1.03–1.24)	1.14* (1.04–1.25)
Missing	0.96 (0.94–1.44)	0.95 (0.77–1.17)	0.96 (0.78–1.19)	0.95 (0.77–1.18)	0.95 (0.77–1.18)	0.94 (0.77–1.17)	0.96 (0.77–1.18)
Instrumental support (ref. available)							
Not available	1.19* (1.10–1.29)	1.19* (1.10–1.29)	1.19* (1.10–1.29)	1.19* (1.11–1.29)	1.19* (1.10–1.29)	1.19* (1.11–1.29)	1.20* (1.11–1.29)
Missing	1.16 (0.94–1.45)	1.17 (0.94–1.44)	1.17 (0.94–1.45)	1.16 (0.94–1.44)	1.16 (0.77–1.18)	1.16 (0.94–1.43)	1.17 (0.95–1.44)
Frequency of meeting friends (ref. almost every day)							
2–3 times/week	1.01 (0.91–1.12)	1.01 (0.91–1.13)	1.01 (0.91–1.13)	1.01 (0.91–1.12)	1.01 (0.91–1.12)	1.01 (0.91–1.12)	1.01 (0.90–1.11)
About once/week	1.00 (0.89–1.11)	1.00 (0.90–1.12)	0.99 (0.89–1.11)	1.00 (0.89–1.12)	0.99 (0.89–1.12)	1.00 (0.89–1.12)	0.99 (0.89–1.11)
1–2 times/month	1.02 (0.91–1.13)	1.02 (0.91–1.13)	1.00 (0.90–1.12)	1.02 (0.92–1.14)	1.02 (0.91–1.13)	1.02 (0.92–1.14)	1.01 (0.91–1.13)
A few times a year or less	1.05 (0.94–1.17)	1.03 (0.93–1.15)	1.02 (0.92–1.14)	1.06 (0.95–1.18)	1.05 (0.95–1.17)	1.06 (0.95–1.17)	1.04 (0.94–1.16)
Missing	1.08 (0.93–1.26)	1.07 (0.92–1.25)	1.09 (0.93–1.26)	1.08 (0.93–1.26)	1.08 (0.93–1.25)	1.08 (0.93–1.25)	1.09 (0.94–1.26)
IADL (ref. not decline)							
Decline	1.23* (1.15–1.32)	1.22* (1.14–1.31)	1.23* (1.14–1.31)	1.24* (1.16–1.33)	1.23* (1.15–1.32)	1.24* (1.16–1.33)	1.24* (1.16–1.33)
Missing	1.20* (1.08–1.34)	1.20* (1.07–1.33)	1.20* (1.08–1.34)	1.21* (1.08–1.34)	1.20* (1.08–1.34)	1.20* (1.08–1.34)	1.21* (1.09–1.34)

HR: Hazard ratio; CI: confidence interval; ref: reference. *$p < 0.05$.

Model 1: Crude model + sex, age, equivalent income, educational attainment, marital status, and self-reported medical conditions. Model 2: Model 1 + smoking, alcohol consumption, walking time (per. day), frequency of going outdoors, depression, emotional support, instrumental support, frequency of meeting friends, and IADL.
